# Contributions of sex, depression, and cognition on brain connectivity dynamics in Parkinson’s disease

**DOI:** 10.1038/s41531-021-00257-9

**Published:** 2021-12-16

**Authors:** Maria Diez-Cirarda, Iñigo Gabilondo, Naroa Ibarretxe-Bilbao, Juan Carlos Gómez-Esteban, Jinhee Kim, Olaia Lucas-Jiménez, Rocio Del Pino, Javier Peña, Natalia Ojeda, Alexander Mihaescu, Mikaeel Valli, Maria Angeles Acera, Alberto Cabrera-Zubizarreta, Maria Angeles Gómez-Beldarrain, Antonio P. Strafella

**Affiliations:** 1 Brain Health Imaging Centre, Campbell Family Mental Health Research Institute, Centre for Addiction and Mental Health, University of Toronto, Toronto, ON Canada; 2 E.J. Safra Parkinson Disease Program & Movement Disorder Unit, Neurology Division; Krembil Brain Institute, University Health Network, University of Toronto, Toronto, ON Canada; 3 Neurodegenerative Diseases Group, Biocruces Bizkaia Health Research Institute, Barakaldo, Spain; 4 IKERBASQUE, The Basque Foundation for Science, Bilbao, Spain; 5 Department of Psychology, Faculty of Health Sciences, University of Deusto, Bilbao, Spain; 6 Neurology Department, Cruces University Hospital, Barakaldo, Spain; 7 OSATEK, MR Unit, Hospital of Galdakao, Galdakao, Bilbao Spain; 8 Neurology Service, Hospital of Galdakao, Galdakao, Bilbao Spain

**Keywords:** Parkinson's disease, Neurological manifestations

## Abstract

Alterations in time-varying functional connectivity (FC) have been found in Parkinson’s disease (PD) patients. To date, very little is known about the influence of sex on brain FC in PD patients and how this could be related to disease severity. The first objective was to evaluate the influence of sex on dynamic FC characteristics in PD patients and healthy controls (HC), while the second aim was to investigate the temporal patterns of dynamic connectivity related to PD motor and non-motor symptoms. Ninety-nine PD patients and sixty-two HC underwent a neuropsychological and clinical assessment. Rs-fMRI and T1-weighted MRI were also acquired. Dynamic FC analyses were performed in the GIFT toolbox. Dynamic FC analyses identified two States: State I, characterized by within-network positive coupling; and State II that showed between-network connectivity, mostly involving somatomotor and visual networks. Sex differences were found in dynamic indexes in HC but these differences were not observed in PD. Hierarchical clustering analysis identified three phenotypically distinct PD subgroups: (1) Subgroup A was characterized by mild motor symptoms; (2) Subgroup B was characterized by depressive and motor symptoms; (3) Subgroup C was characterized by cognitive and motor symptoms. Results revealed that changes in the temporal properties of connectivity were related to the motor/non-motor outcomes of PD severity. Findings suggest that while in HC sex differences may play a certain role in dynamic connectivity patterns, in PD patients, these effects may be overcome by the neurodegenerative process. Changes in the temporal properties of connectivity in PD were mainly related to the clinical markers of PD severity.

## Introduction

In the last few years, functional connectivity (FC) during resting-state functional magnetic resonance imaging (rs-fMRI) has been used in Parkinson’s disease (PD) to study the neural mechanisms of various complications, such as cognitive impairment^1–3^ and depression^4–7^. Recently, a number of studies have applied time-varying analysis methods for brain connectivity, also known as dynamic FC, in diverse populations^8,9^. These chronnectome techniques assume that brain connectivity is not static but changes over time, and aim to identify dynamic and reoccurring patterns of connectivity^10^. Alterations in dynamic FC have been found in major depression^11,12^, psychiatric disorders^13,14^, and neurodegenerative diseases, including Alzheimer’s disease and PD^15–18^. Previous studies in PD have identified two recurring FC States^16,19,20^. Dynamic FC alterations have been reported in the disease, showing changes in the number of *state transitions*, and reduced time spent in the sparsely connected state, linked to an increased time spent in the strongly between-network connected state^16^. These alterations have shown relationships with motor symptoms and various degrees of cognitive impairment^16,19,20^. Increased motor complications were related to the increased number of *state transitions* and associated with reduced time spent in the within-network connected state^16^. Additionally, the presence of cognitive impairment in PD was also related to changes in *state transitions* and altered dwell time in dynamic states^19,20^. However, little is known about other highly prevalent and disabling non-motor symptoms in PD, such as depression^21^, and its association with the intrinsic dynamic patterns of brain connectivity.

Another factor that needs to be taken into account when analyzing the relationship between PD symptomatology and brain connectivity is sex differences. Previous literature has reported sex differences in PD motor and non-motor symptoms^22–25^. The analysis of the differences in brain FC between sexes could help to understand the mechanisms that explain the clinical differences between females and males with PD, but literature on this matter is very scarce. One previous study in de novo PD investigated sex differences in resting-state brain static FC in drug-naïve patients^26^. Results showed no significant differences in whole-brain connectivity, including the somatomotor (SM) network, default-mode network (DMN), dorsal and ventral attention networks, and frontoparietal network, but revealed slight differences with region-of-interest analysis and other neurophysiological recordings in the basal ganglia and SM network^26^.

On the contrary, sex differences have shown an impact on resting-state functional network connectivity in healthy adults. The literature suggests that female adults may show stronger connectivity within the DMN, while male adults present instead stronger connectivity patterns within and between the SM and visual (VIS) networks^27–30^. A recent dynamic FC study in healthy subjects also revealed a more unstable pattern of connectivity in males than in females^31^. These sex differences in healthy subjects in their brain static and dynamic FC have been associated with trends in behavioral differences^31,32^.

Increasing our knowledge about the contributions of sex in brain connectivity in PD could help to better understand PD heterogeneity and its association with motor and non-motor symptoms. In light of this, the objectives of the present study were twofold. The first aim was to evaluate for the first time the influence of sex on dynamic FC characteristics in PD patients and healthy controls (HC). The second objective was to investigate the temporal patterns of dynamic connectivity associated with PD severity, considering motor symptoms, depression, and cognition.

## Results

### Sociodemographic and clinical characteristics for HC and PD groups

Demographic and clinical characteristics are shown in Table 1. No significant sex differences were found in HC and PD participants for cognition or clinical features between PD males and females. Years of education and the presence of depression were included as covariates in the following analyses. Cognition and depression were also tested with a two-way multivariate analysis of covariance (MANCOVA). Significant results were found for group effect on cognition [assessed with the Montreal Cognitive Assessment (MoCA)] (*F* = 15.758; *p* < 0.001) and depressive symptoms [assessed with the 15-item Geriatric Depression Scale (GDS-15)] (*F* = 20.734; *p* < 0.001), showing PD patients greater cognitive impairment and depressive symptoms compared to HC. GroupxSex interaction was not significant in MoCA (*F* = 0.641; *p* = 0.425) or GDS-15 (*F* = 0.214; *p* = 0.645).

**Table 1:** Sex differences in sociodemographic and clinical characteristics in HC and PD patients.

	PD sample (*n* = 99)				HC sample (*n* = 62)			
	PD Male (*n* = 69)	PD Female (*n* = 30)	*F*	*p*	HC Male (n = 31)	HC Female (*n* = 31)	*F*	*p*
**Age (years)**	63.36 (8.16)	65.48 (6.85)	1. 546	0.217	64.38 (7.90)	62.20 (8.42)	1.109	0.297
**Education (years)**	13.14 (4.92)	10.73 (3.90)	5.637	0.020	13.23 (4.22)	12.42 (4.80)	0.492	0.486
**MoCA**	24.53 (3.53)	24.20 (3.65)	0.186	0.668	26.10 (3.00)	26.87 (2.12)	1.319	0.256
**GDS-15**	2.82 (2.52)	2.54 (2.66)	0.241	0.625	1.31 (1.80)	0.63 (1.02)	3.203	0.079
**Disease duration**	6.01 (4.86)	7.24 (4.54)	1.374	0.244	–	–	–	–
**UPDRS III**	26.52 (10.65)	24.00 (12.33)	1.063	0.305	–	–	–	–
**Hoehn and Yahr**	1.97 (0.45)	2.06 (0.50)	0.871	0.353	–	–	–	–
**LEDD**	622.57 (373.62)	705.45 (491.58)	0.844	0.360	–	–	–	–

Values are shown as mean (standard deviation).

*HC* healthy controls, *PD* Parkinson’s disease, *MoCA* Montreal cognitive assessment, *GDS-15* geriatric depression scale, *UPDRS III* unified PD rating scale, motor subscale, *LEDD* levodopa equivalent daily dose.

### Dynamic FC states

**Fig. 1:**
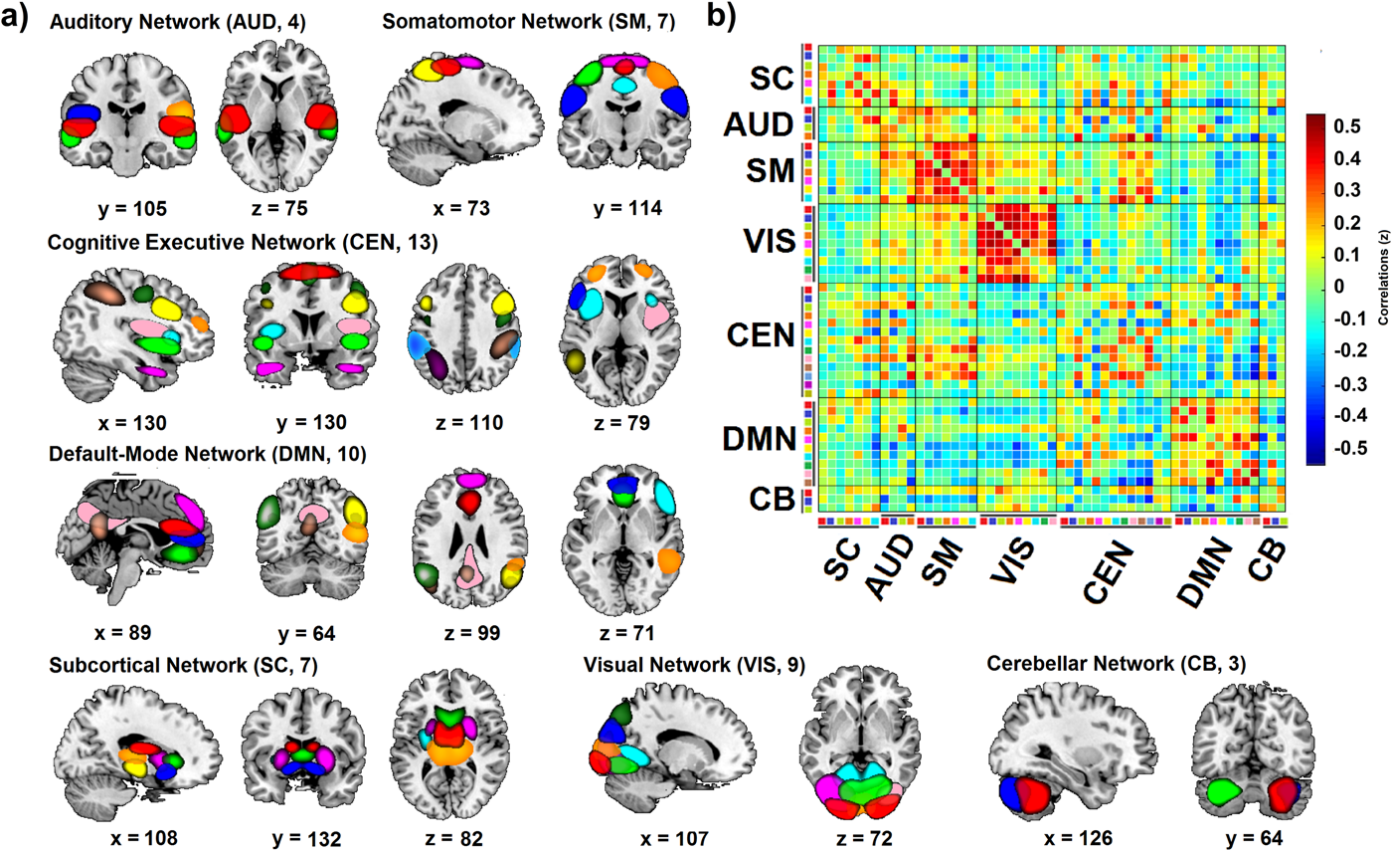
Representation of the ICs grouped in each intrinsic connectivity network. Representation of the ICs grouped in each Network. **a** Spatial representation of the 53 ICs identified by ICA grouped in functional networks. **b** Group averaged static FC matrix between the 53 ICs. Correlation values between ICs represent the Fisher’s z-transformed Pearson correlation coefficient. Color-coded legend of each component matches colors between **a** and **b**. More information about the ICs is shown in Supplementary Table 2.

**Fig. 2:**
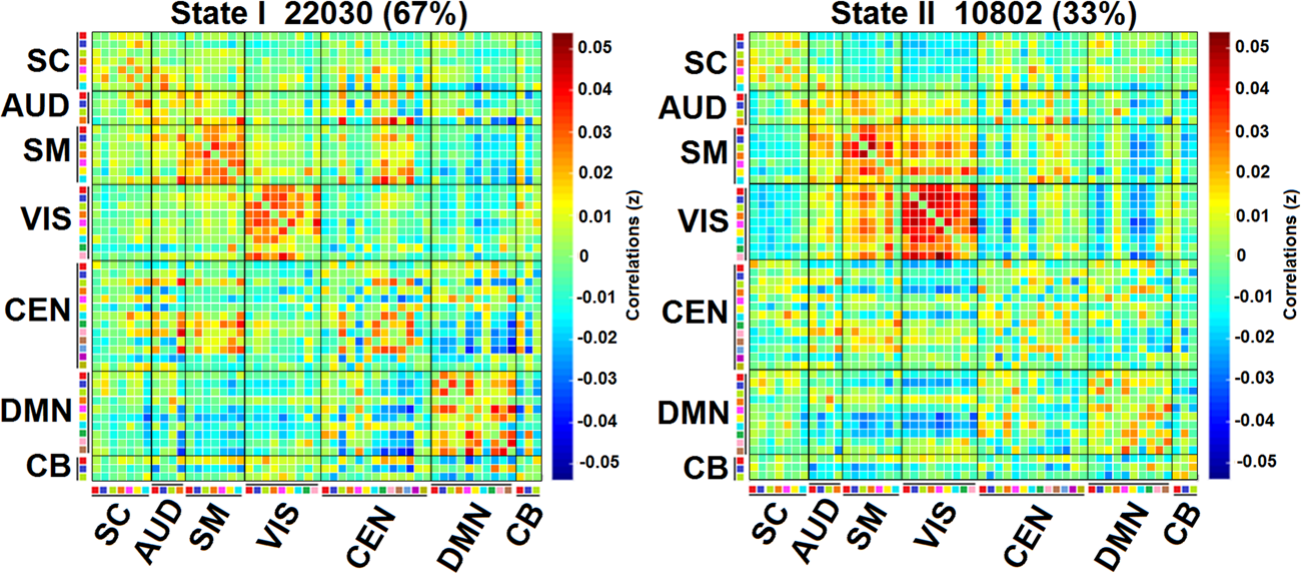
Cluster centroids for each dynamic State. Cluster centroids for each dynamic FC State. Specific ICs colors within each network corresponds to the same colors in Fig. 1 and Supplementary Table 2 of the manuscript. The total number of windows is specified in each State I and II. The percentage indicates the occurrence rate. SC subcortical network, AUD auditory network, SM somatomotor network, VIS visual network, CEN cognitive executive network, DMN default-mode network, CB cerebellar network.

Independent component analysis (ICA) resulted in 53 independent components (ICs) grouped in seven networks (Fig. 1). Dynamic FC analysis identified two highly structured FC States in all participants (Fig. 2). Results showed a more frequent State I (67%) with sparse positive coupling mainly within networks [e.g., DMN, SM, VIS, and central-executive network (CEN)], and a less frequent State II (33%) with stronger FC between networks. The latter between-network connectivity pattern included both negative and positive coupling, mainly in the posterior-central regions between the SM and VIS networks.

### Dynamic FC States and sex differences in HC and PD

**Fig. 3:**
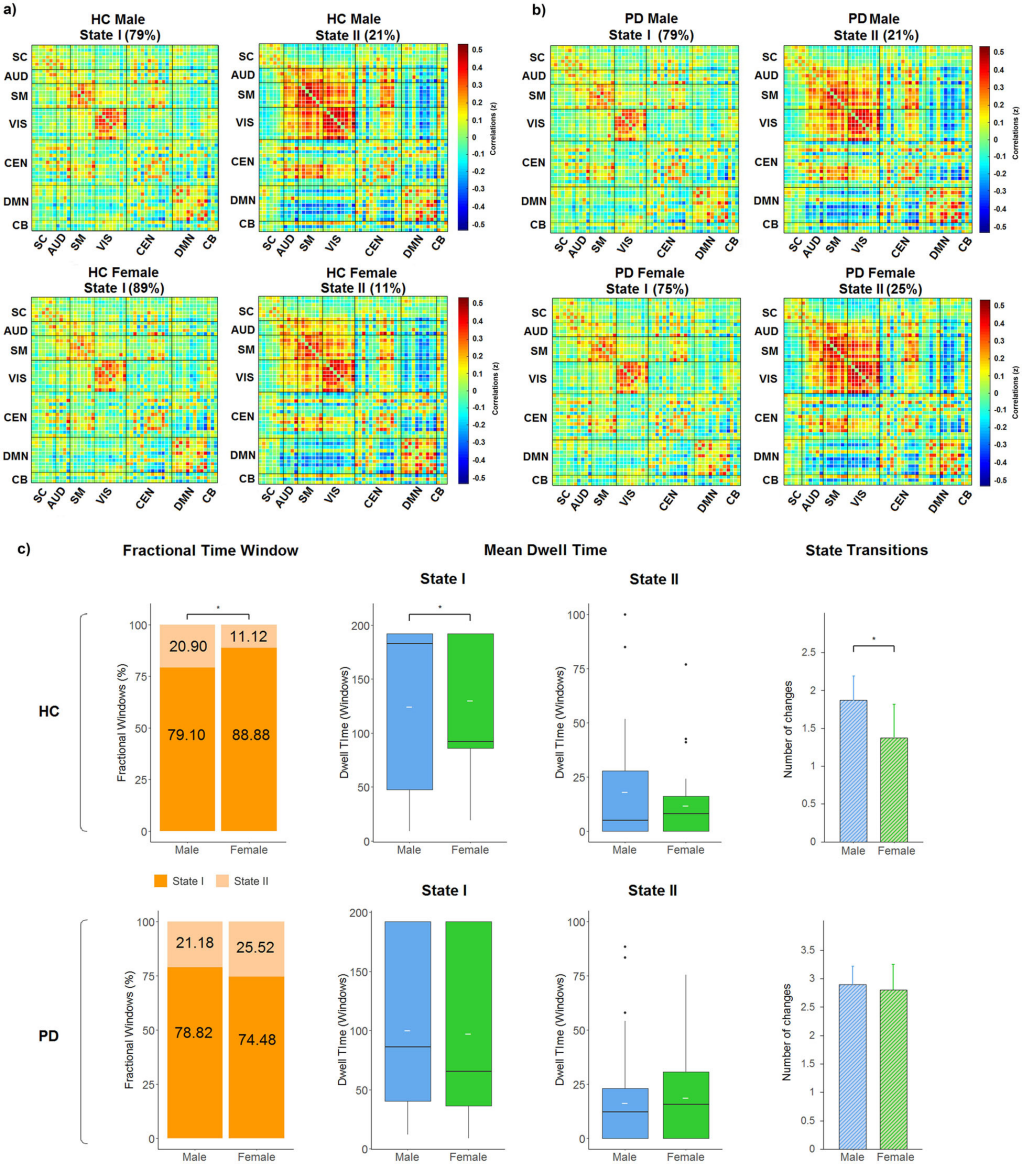
Dynamic FC States and sex differences in HC and PD. **a**, **b** Group centroids for each FC State per group. The percentage indicates the occurrence rate. **c** Sex differences in dynamic FC indexes in HC and PD groups. A *fractional time window* is represented by the mean percentage in each State; *Mean dwell time* is represented with boxplots. The median and mean are represented by black and white lines, respectively. *State transitions* are represented with mean and error bars. *p < 0.05. HC healthy controls, PD Parkinson’s disease, SC subcortical network, AUD auditory network, SM somatomotor network, VIS visual network, CEN cognitive executive network, DMN default-mode network, CB cerebellar network.

The connectivity patterns in each State for male and female PD and HC subgroups are shown in Fig. 3a, b. In both HC and PD groups, males and females showed a more frequent State I and a less frequent State II with stronger FC between networks. Sex differences in connectivity in State I or State II did not survive false discovery rate (FDR) correction.

Two-way MANCOVA showed that there was a significant GroupxSex interaction effect in the temporal properties of connectivity. Specifically, we found a significant GroupxSex interaction effect in *fractional time window* in State I and State II (*F* = 3.020; *p* = 0.020), *mean dwell time* in State I (*F* = 4.269; *p* = 0.003), and *state transitions* (*F* = 4.287; *p* = 0.003).

Within-group analysis revealed sex differences in dynamic indexes in HC. HC females significantly spent more time (*fractional time window*) in the within-network connected State I compared to HC males [females: mean ± standard deviation (SD) = 88.88 ± 20.69%; males: mean ± SD = 79.10 ± 28.66%] (Fig. 3c), suggesting that the female group exhibited a higher proportion of time spent in the more frequent State I with sparse coupling mainly within networks, while HC males tended to stay in State II with stronger between-network connectivity (mostly between the SM and VIS networks). In addition, HC females showed increased *mean dwell time* in State I (mean ± SD = 130.20 ± 63.75) compared to HC males (mean ± SD = 124.68 ± 76.25) (*F* = 4.538; *p* = 0.015), which indicates that females tend to spend more time in the within-network connected state before switching to the other state. Significant differences in *state transitions* were found in HC males compared to HC females (males: mean ± SD = 1.87 ± 2.21; females: mean ± SD = 1.38 ± 1.56) (*F* = 4.567; *p* = 0.015) (Fig. 3c), suggesting an increased number of transitions between States in the male group. Overall, these observations suggested significant sex differences in HC with greater stability of the sparse coupling within-network FC (State I) in females. In contrast, the male sample expressed increased transitions between states with less stability and a tendency for a longer length of stay in the between-network FC (State II).

There were no significant differences in dynamic indexes when comparing males and females in the PD sample. No specific differences were present in any dynamic index, suggesting that both PD males and females spent a similar time in the within- and between-network connected states. Overall, these observations suggested that sex was probably not a contributing factor in the time-varying FC changes in PD patients; thus, we aimed to investigate other potential contributing factors of PD on the temporal properties of connectivity.

### Demographic and clinical characteristics of PD subgroups

**Fig. 4:**
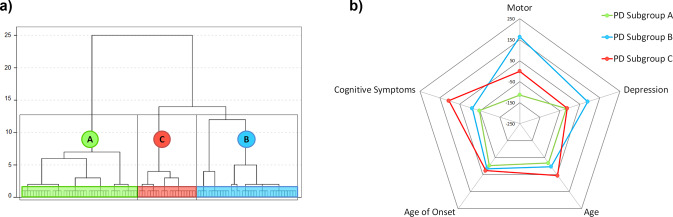
Dendrogram of clustering analysis (a) and visual representation (b) of PD subgroup symptomatology. **a** Dendrogram of clustering analysis of PD patients divided according to the motor and non-motor symptomatology; **b** Visual representation of each PD subgroup. HCA was performed with motor, cognitive, and depression scores. Age and age of disease onset are used as references for sociodemographic and clinical aspects. Scores are shown in (*z*-scores*100) for visualization purposes. Cognitive symptoms represented by MoCA scores (*z*-scores, inverse values); Motor = Composite score created of the UPDRS III and Hoehn & Yahr for visual representation (*z*-score). Depression = Geriatric Depression Scale (*z*-score). PD Parkinson’s disease.

To accurately classify the patients based on the severity of symptoms, including both motor and non-motor symptoms, we used hierarchical agglomerative clustering analysis. The hierarchical clustering analysis identified three distinct PD subgroups based on motor and non-motor symptoms (Table 2 and Fig. 4). Principal component analysis (PCA) validated the PD subgroup classification (Supplementary Fig. 1).

**Table 2:** Sociodemographic and clinical characteristics of PD subgroups and HC.

	HC (*n* = 62)	PD subgroup A (mild subtype) (*n* = 42)	PD subgroup B (depression dominant) (*n* = 36)	PD subgroup C (cognitive dominant) (*n* = 21)	*F*	*p*
**Age (years)**	63.29 (8.17)	62.02 (8.24)	64.03 (7.74)	67.93 (5.54)	2.779	0.043^a^
**Sex (male/female)**	31/31	29/13	26/10	14/7	*χ*^2^ = 6.472	0.091
**Education (years)**	12.82 (4.5)	13.74 (4.42)	11.69 (4.78)	11.00 (4.87)	2.217	0.088
**MoCA**	26.50 (2.59)	26.30 (2.63) [15–30]	24.75 (2.88) [15–30]	20.14 (2.49) [13–23]	32.839	<0.001^b^
**GDS-15**	0.97 (1.49)	1.63 (1.83) [scores: 0–6]	4.22 (2.92) [scores: 0–11]	2.39 (1.87) [scores: 0–7]	20.231	<0.001^c^
**FD mean**	0.17 (0.07)	0.16 (0.07)	0.20 (0.08)	0.20 (0.07)	2.436	0.067
**Age at disease onset**	–	57.02 (7.93)	56.33 (7.39)	59.35 (9.78)	0.937	0.395
**UPDRS III**	–	19.00 (8.10) [2–41]	34.81 (9.03) [20–57]	23.76 (9.40) [8–41]	32.471	<0.001^d^
**Hoehn and Yahr**	–	1.70 (0.38) [1.0–2.0]	2.34 (0.42) [1.5–3]	2.00 (0.22) [1.5–2.5]	*χ*^2^ = 49.252	<0.001^e^
**LEDD**	–	558.72 (312.22)	620.25 (355.64)	872.66 (583.01)	4.486	0.014^f^

Values are shown as mean (standard deviation).

*HC* healthy controls, *PD* Parkinson’s disease, *MoCA* Montreal cognitive assessment, *GDS-15* geriatric depression scale, *FD* framewise displacement, *UPDRS III* unified PD rating scale, motor subscale, *LEDD* levodopa equivalent daily dose.

^a^Significant post hoc differences showing PD subgroup C > PD subgroup A (*p* = 0.027).

^b^Significant post hoc differences showing PD subgroup C < HC (*p* < 0.001), PD subgroup A (*p* < 0.001) and PD subgroup B (*p* < 0.001); and PD subgroup B < HC (*p* = 0.011).

^c^Significant post hoc differences showing PD subgroup B > HC (*p* < 0.001), PD subgroup A (*p* < 0.001) and PD subgroup C (*p* = 0.007); and PD subgroup C < HC (*p* = 0.032).

^d^Significant post hoc differences showing PD subgroup B > PD subgroup A (*p* < 0.001) and PD subgroup C (*p* < 0.001).

^e^Significant post hoc differences between all groups (*p* < 0.001).

^f^Significant post hoc differences showing PD subgroup C < PD subgroup A (*p* = 0.011).

PD subgroup A (mild subtype) was characterized by mild motor symptoms [Unified PD Rating Scale (UPDRS) III = 19.00 ± 8.10]. PD subgroup B (depression dominant subtype) was characterized mainly by motor symptoms (UPDRS III = 34.81 ± 9.03), the presence of depressive symptoms [43.3% had a GDS-15 > 5, and the rest had depressive symptoms], and slight cognitive differences with HC. PD subgroup C (cognitive dominant subtype) was characterized mainly by the presence of moderate motor symptoms (UPDRS III = 23.76 ± 9.40), pronounced cognitive impairment (MoCA = 20.14 ± 2.49), with slight depression score differences compared to HC. Chi-squared analysis revealed no significant differences in the percentage of males and females between PD subgroups and HC (see Table 2).

### Dynamic FC States in PD subgroups and HC

Connectivity patterns in each State for each PD subgroup and HC are visualized in Supplementary Fig. 2, showing that all PD subgroups had a more frequent State I with sparse positive coupling mainly within networks (e.g., DMN, SM, VIS, and CEN) and a less frequent State II with stronger FC between networks. Differences in connectivity in State I or State II in PD subgroups compared to HC did not survive FDR correction.

PD subgroups exhibited variations in the temporal properties of connectivity compared to HC. MANCOVA revealed significant differences between groups in *fractional time window* (State I and State II) (*F* = 3.153; *p* = 0.027), *mean dwell time* in State I (*F* = 3.369; *p* = 0.020), and *state transitions* (*F* = 5.891; *p* < 0.001).

**Fig. 5:**
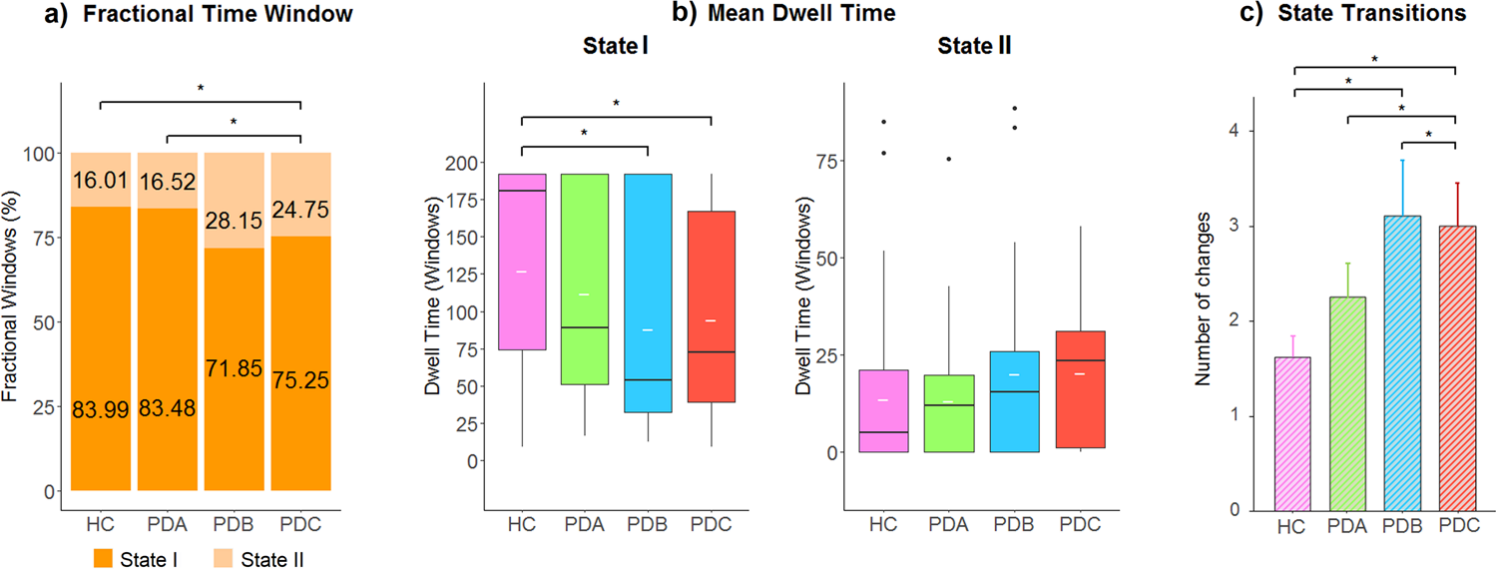
Dynamic FC differences in PD subgroups and HC. Post hoc significant differences between groups. *p < 0.05; **a** *Fractional time window* is represented by the mean percentage in each state; **b** *Mean dwell time* is represented with boxplots. The median and mean are represented by black and white lines, respectively. **c** *State transitions* are represented with mean and error bars. PD Parkinson’s disease, HC healthy controls.

In detail, in the HC group, the *fractional time window* or total occurrences of State I were more frequently observed (mean ± SD = 83.99 ± 25.27%) than of State II (mean ± SD = 16.01 ± 25.27%), and a similar percentage was observed in the PD subgroup A (mean ± SD = 83.48 ± 22.59% and 16.52 ± 22.59%). Instead, in the PD subgroups B and C, State I occurred less frequently (Subgroup B: mean ± SD = 71.85 ± 29.20%; Subgroup C: mean ± SD = 75.25 ± 25.88%), while State II re-occurred at a higher rate (Subgroup B: mean ± SD = 28.15 ± 29.20% and subgroup C: mean ± SD = 24.75 ± 25.88%) (Fig. 5a). Post hoc analyses revealed significant differences in *fractional time window* between PD subgroup C and HC (*p* = 0.011) and PD subgroup A (*p* = 0.010) (Fig. 5a).

In addition, *mean dwell time* in State I was shorter in the more symptomatic PD subgroups (PD subgroup B: mean ± SD = 88.09 ± 71.98%; PD subgroup C: mean ± SD = 94.14 ± 68.91%) compared to HC (mean ± SD = 127.44 ± 69.76%) and PD subgroup A (mean ± SD = 111.74 ± 69.73%). Post hoc analyses revealed significant differences in PD subgroup B compared to HC (*p* = 0.035), and in PD subgroup C compared to HC (*p* = 0.005), suggesting a shortened length of stay in the State with within-network connectivity (Fig. 5b).

Group differences were also found in the number of transitions between States. PD subgroups B and C showed significantly increased *state transitions* compared to HC (mean ± SD = 1.62 ± 1.91) [PD subgroup B (mean ± SD = 3.11 ± 2.52, *p* = 0.005); PD subgroup C (mean ± SD = 3.00 ± 2.58, *p* < 0.001)]. Moreover, PD subgroup C showed differences in *state transitions* compared to PD subgroup B (*p* = 0.022) and PD subgroup A (*p* = 0.032) (Fig. 5c). Overall, these observations suggest that the stability of the connectivity is altered in symptomatic PD subgroups.

Finally, we analyzed the sex differences within each PD subgroup, and we found no significant differences in dynamic indexes between males and females in any of the PD subgroups A, B, or C (see “supplementary results” in supplementary materials).

### Relationships between PD symptomatology and temporal properties of connectivity

Correlation analyses were performed to evaluate the relationships between dynamic FC indexes and PD motor and non-motor symptoms. *Z*-scores of the UPDRS III, Hoehn & Yahr, MoCA, and depression scores were combined into one variable that represented PD symptomatology. Results showed significant associations between PD symptomatology and *fractional time window* in State I (*r* = −0.196; *p* = 0.028), State II (*r* = 0.196; *p* = 0.028), *mean dwell time* in State I (*r* = −0.185; *p* = 0.035), and *state transitions* (*r* = 0.175; *p* = 0.044). These results suggest that greater PD symptomatology was related to reduced time spent in the within-network connected State I, while increased time spent in the between-network connected State II, accompanied by a more unstable pattern of connectivity.

## Discussion

This study aimed to analyze the effect of sex and PD-related motor and non-motor manifestations in time-varying FC patterns of the brain. The most compelling finding was that while in healthy adults sex differences influenced dynamic FC patterns, in PD patients sex was not a contributing factor in the time-varying FC changes. In these parkinsonian patients, changes in the temporal properties of connectivity were mainly related to motor and non-motor symptoms linked to disease severity.

Dynamic FC analysis identified two recurring FC States. A more frequent State (State I) with sparse within-network connectivity, involving mainly the DMN, CEN, VIS, and SM networks, with stronger activity in frontal and posterior areas; and a less frequent State (State II) characterized by stronger between-network connectivity, mostly between the SM and VIS networks, showing stronger activity in posterior brain areas. Previous dynamic studies in PD also found two different dynamic FC States with similar characteristics^16,19,20^.

In the HC group, sex differences were found between males and females in the temporal properties of dynamic FC. More specifically, HC females spent more time (i.e., *fractional time window*) in the within-network connected State (State I), and spent less time in the between-network connected State (State II) compared to HC males. Similar results were found in *mean dwell time*, suggesting a reduced length of stay in the within-network connected State in HC males compared to HC females. Interestingly, State I was characterized by stronger within-network connectivity including the DMN, while State II was characterized by stronger between-network SM–VIS network connectivity. These results are consistent with the previous brain static FC studies in HC, showing greater FC mostly within the DMN in HC females, and greater FC within the SM network and between SM–VIS network connectivity in HC males^27–30,33^. The DMN has been related to social cognition^34^ and semantic memory^35^, while the SM and VIS networks have been related to visuospatial and sensorimotor skills^36^. The coordination between sensory and motor networks has been associated with motor learning^37^. One possible hypothesis could suggest a relationship between these brain dynamic FC differences and results from previous studies that show tendencies in behavioral differences between females and males, suggesting that males tend to score higher in motor and visuospatial skills, and women tend to have higher scores in social cognition and memory^32^. Moreover, HC males in the present study showed a more unstable pattern of connectivity (i.e., increased changes between states) compared to HC females. Similarly, in a recent dynamic FC study, HC males showed increased brain connectivity dynamism compared to females, which correlated with mental rotation performance and response inhibition^31^.

On the contrary, when evaluating sex differences among PD patients, these differences in the temporal properties of dynamic FC were not observed. In the present study, we analyzed whole-brain time-varying FC in PD patients with a mean disease duration of 6–7 years. These results are in line with observations from a previous PD study analyzing sex-related brain differences in de novo patients^26^. In that study, authors investigated whole-brain static FC differences and did not detect different patterns of connectivity at the cortical level between PD males and females^26^. However, the region-specific analysis showed some sex differences only in the SM network (including the basal ganglia and cortical areas) with increased values in female PD patients compared to male PD patients^26^. Another study found that female PD patients presented with higher [^123^I]FP-CIT striatal binding compared to males with an early onset of PD symptoms^38^. Altogether, these results suggest that sex differences in PD may be detectable at very early stages of the disease in specific brain areas, but as the neurodegeneration progresses and worsens, sex differences may no longer have an effect on brain connectivity and may be overcome by the neurodegenerative process. This hypothesis is reinforced by the correlational results from the present study showing significant associations between the altered patterns of dynamic connectivity and PD-related symptomatology. Another possible hypothesis is that sex differences in PD brain connectivity might be detectable in specific brain regions (i.e., subcortical) or specific brain circuits. In the present study, we did not find significant differences in connectivity in State I or State II between PD males and females. Further research is needed to better understand this subject.

The clustering analysis identified three highly symptom-based differentiated PD groups: PD subgroup A, which included patients with less severe motor symptoms and no significant non-motor complications; PD subgroup B, represented by patients with motor symptoms and predominantly depressive symptomatology, showing slight cognitive differences compared to healthy subjects; and PD subgroup C, which included patients with motor symptoms and predominantly cognitive impairment, but with slight differences in depressive symptoms compared to healthy subjects. Interestingly, the literature has suggested different non-motor subtypes of PD, including depression-dominant and cognitive-dominant subtypes with related routes of spread of the pathophysiological process^39^.

Results revealed that the most significant changes in dynamic FC in PD patients were mainly related to the presence of motor and non-motor complications. An observation worth highlighting is that both symptomatic groups (depression-dominant and cognitive-dominant subtypes) showed the same directionality in the alterations in temporal connectivity patterns. In detail, both of the more symptomatic PD subgroups showed reduced time spent (8–12% of the total time) in the more segregated State (State I) and proportionally increased total time spent in the stronger between-network connected State (State II). Reduced time spent in the sparsely connected State and proportionally increased time spent in the interconnected State has been previously described in symptomatic PD^16^. Conversely, PD patients with less motor severity and a lack of non-motor symptoms (PD subgroup A) showed a similar pattern of dynamic FC to HC, which may suggest that dynamic alterations may be more accentuated with disease progression.

As noted above, PD patients with depression-dominant subtype showed significant dynamic alterations with strongly reduced time spent in the sparsely connected State I, involving the DMN, CEN, VIS, and SM networks, and proportionally increased time spent in the highly between-network connected State II. Previous studies evaluated the FC variability across sliding windows and found that patients with major depressive disorder showed increased variability in regions of the CEN and DMN, including the dorsolateral prefrontal cortex, anterior cingulate cortex, amygdala, and hippocampal area^12^. Interestingly, in our study, PD patients with depressive symptoms showed increased variability (i.e., *State transitions*) between State I (2% of the strongest connections included the CEN within-network) and State II (with no strong within-network connectivity in the CEN). Thus, the present results reinforce the observation that depressive symptoms in PD patients are associated with a more unstable pattern of connectivity mostly in the executive network with a greater tendency to prefer the between-network connected State (i.e., reduced functional segregation).

Moreover, PD patients with the cognitive-dominant subtype also showed alterations in the temporal patterns of connectivity. Results showed that PD patients with cognitive deficits increased the time spent in State II with stronger between-network connectivity compared to PD patients with normal cognition and HC. That is, the early presence of cognitive deficits may be related to reduced within-network brain activity mainly in the DMN, SM, VIS, and CEN and increased connectivity between the SM and VIS networks (i.e., reduced functional segregation). Interestingly, State II was characterized by a reduced negative correlation between the DMN and CEN compared to State I, and this was previously related to PD patients with mild cognitive impairment^1^. Findings also identified an altered number of transitions between states in PD patients with cognitive impairment compared to healthy adults. Some previous PD studies have demonstrated similar results, such as increased time spent in the between-network connected state^16,20^ or increased *state transitions*^20^, while these differences were not found in another study of PD patients with dementia^19^. Interestingly, PD patients with dementia were reported with a reduced number of changes between States and decreased time spent in the between-network connected State compared to PD patients with cognitive impairment and normal cognition^19^. These observations may suggest that early cognitive symptoms show slight time-varying connectivity changes in PD, possibly because of likely compensatory changes, which may change and decrease when symptoms become more severe and compensatory mechanisms fail. Increased connectivity between the SM and VIS networks has been related to motor learning^37^ and previously found in PD as a compensatory mechanism^40^. One hypothesis goes along with a possible network reorganization to overcome the PD-related striato-cortical loop deficits. This reorganization may be effective only temporarily until the severity of PD symptoms worsens and the compensatory mechanism starts failing^41–43^.

Altogether, these findings seem to suggest that within-network connectivity (i.e., functional segregation) is the most prominent FC pattern during resting-state in HC and in early PD patients. However, with the progression of disease severity, PD patients tend to express reduced functional neural segregation by switching to between-network connectivity (mostly between the SM and VIS networks), suggesting a possible network reorganization. Increased between-network coupling during resting-state has been related to higher-frequency oscillations in the “alpha” and “beta” bands^44^. Beta oscillations showed positive correlations with activity in the bilateral temporo-parietal junction and VIS, auditory (AUD), and SM networks. This increment of between-network synchronization in PD has been associated with disease progression^45,46^ and suggested to be a compensatory mechanism to overcome the PD-related striato-cortical loop deficits^41–43^. The present study did not detect differences in motor, cognitive, or depressive symptoms between PD males and females. We speculate that the use of global screening scales (e.g., UPDRS and MoCA) may not be able to detect specific differences, as already seen in previous PD studies using similar measures^24,47^.

Some limitations of the study should be considered. Cognitive deficits were assessed with the MoCA. The MoCA score gives an overall estimation of cognitive performance; thus, it would be better to evaluate cognition with a more comprehensive assessment (level II) as suggested by the Movement Disorder Society task force criteria for mild cognitive impairment in PD^48^. In addition, there was an unbalanced of males and females between groups. Finally, depressive symptoms sometimes coexist with symptoms of anxiety and apathy in PD^49^. Future studies should include evaluations of these other non-motor symptoms in order to assess and differentiate their relevance and relationship with dynamic alterations. From a methodological point of view, TE from the resting-state of one of the MRI acquisition center was slightly lower.

In conclusion, this study evaluated the influence of sex on brain temporal FC patterns in PD and HC and provided several new observations. One of the interesting observations was that while in healthy adults, sex-related differences played a certain role in dynamic connectivity patterns, these may no longer have an effect on PD brain connectivity as the neurodegenerative process worsens. In these parkinsonian patients, changes in the temporal properties of dynamic connectivity were mainly related to motor and non-motor symptoms linked to disease severity and suggested a possible mechanism of compensatory connectivity network reorganization to overcome the PD-related striato-cortical deficits.

## Methods

### Participants

This study included a retrospective pooled database from three research centers: the Biocruces Bizkaia Health Research Institute (Barakaldo, Spain), the University of Deusto (Bilbao, Spain), and the Centre for Addiction and Mental Health (Toronto, Canada). The original pooled database was composed of 129 PD patients and 68 HC. After reviewing MRI data for quality control^50,51^, 24 participants were excluded. In addition, two HC were excluded due to enlarged ventricles. Nine participants were excluded due to participation in the two Spanish research centers, which are in the same province. The database included in this study comprised 100 PD patients and 62 HC. After motion correction during resting-state fMRI, the final sample size was 99 PD patients and 62 HC. At each site, PD patients and HC were recruited. PD patients were enrolled in the study with the following criteria: 1) Fulfill the UK PD Society Brain Bank diagnostic criteria; 2) Hoehn and Yahr disease stage 1–3^52^; and 3) Age range between 45 and 75 years. Exclusion criteria included other neurological or psychiatric disorders, a history of head injury, and incompatibilities with MRI acquisition (e.g., claustrophobia, deep brain stimulation). Disease severity was assessed with the UPDRS III and Hoehn and Yahr stages, along with disease duration and levodopa equivalent daily dose (LEDD) was registered^53^. Cognitive impairment was evaluated with the MoCA^54^ or the Mini-mental State Examination (MMSE)^55^. Depression symptomatology was evaluated with the GDS-15^56^ or the Beck Depression Inventory (BDI II)^57^. MMSE scores were converted into MoCA scores based on multicentre validation^58^. Conversion from the BDI to the GDS was performed as in the previous studies^59^. We found no significant differences in MoCA scores (F = 1.195; *p* = 0.305) or depression scores (F = 0.083; *p* = 0.920) between recruitment centers. Participants in the original studies provided written informed consent approved by the local research ethics committees according to the Declaration of Helsinki. The current retrospective study was approved by the research ethics committee of the Centre for Addiction and Mental Health.

### Neuroimaging acquisition

T1-weighted images were acquired in 3 T scanners, in a sagittal orientation. Acquisition parameters were as follows: Repetition time (TR) = 6.7/7.4 ms; Flip angle 8°/9°; Matrix size = 256 × 256/228 × 218; Slice thickness = 0.9/1.1 mm. The rs-fMRI data were obtained in an axial orientation in an anterior-posterior phase direction using a sequence sensitive to blood oxygen level-dependent (BOLD) contrast. Acquisition parameters were as follows: TR = 2000/2100 ms; Flip angle = 60°/80°; Matrix size = 64 × 64/80 × 79; Slice thickness = 5/3 mm; Volumes = 240/214; Acquisition time = 8′04′′; 7′40″). The complete acquisition parameters are specified in Supplementary Table 1.

Rs-fMRI data were acquired during a so-called resting-state block. Subjects were instructed to neither engage in any particular cognitive nor motor activity, to keep their eyes closed without thinking about anything in particular, and they were told they could not fall asleep. Once the rs-fMRI acquisition terminated, the participant was asked whether they had fallen asleep or not. No patient reported having fallen asleep. Foam padding and headphones were used to limit head movement and reduce scanner noise for the subject. The acquisition site was used as a covariate in all analyses. PD patients were ON medication during the neuroimaging acquisition. All PD patients were receiving stable dopaminergic treatment prior to the assessment. LEDD from each patient was calculated^53^.

### Neuroimaging preprocessing and analysis

Rs-fMRI preprocessing was performed using the CONN toolbox 18.a^60^. All preprocessing steps were conducted using the default preprocessing pipeline, including realignment to the first volume, slice-timing correction (interleaved bottom-up), co-registration to structural data, spatial normalization into the standard MNI space (Montreal Neurological Institute), and finally, a smoothing of Gaussian kernel of 6 mm FWMH was applied. Moreover, the noise was reduced via the anatomical CompCor approach, which extracts principal components from white matter and cerebrospinal fluid time series. These components were added as confounds in the denoising step of the CONN toolbox. The six head motion parameters derived from spatial motion correction were also added as confounds. As recommended, linear detrending and band-pass filtering with a frequency window of 0.008 to 0.09 Hz was performed^61^. For a schematic representation of neuroimaging acquisition, preprocessing, and analysis, see Supplementary Fig. 3.

Group ICA of fMRI Toolbox (GIFT) was used to decompose the data into intrinsic FC networks^62^. First, subject-specific data was reduced to 120 ICs with the principal component reduction. In a second step, the expectation-maximization algorithm was used to perform group-data reduction to 100 ICs, which ensures functional parcellation of cortical and subcortical components^9^. To ensure stability and validity, we repeated the Infomax ICA algorithm 20 times in ICASSO^63^. Then, the GICA back-reconstruction algorithm was used to obtain subject-specific maps and time courses for each IC, as implemented in GIFT software^62^. The spatial correlation values between ICs and the template were used for ICs selection^64^, based on the FC atlas networks^28^ according to these 7 categories: Subcortical (SC), AUD, SM, VIS, CEN (which includes the salience network and language network), DMN, and cerebellar (CB) networks. Components were classified as intrinsic connectivity networks if they exhibited peak activations in gray matter, high correlation values with resting-state networks, and had time courses dominated by low-frequency fluctuations^9^. This process resulted in 53 ICs out of the 100 ICs obtained (see Fig. 1; Supplementary Table 2). After ICs selection, subject-specific spatial maps and times courses were post-processed to remove the remaining noise. This post-processing included detrending, despiking, and a filter cutoff using a fifth-order Butterworth low-pass filter with a high-frequency fluctuation set at 0.15 Hz.

To minimize the impact of head movement in the dynamic connectivity results^8^, strict criteria were applied. Framewise displacement (FD) index was calculated following the published formula^65^. Subjects were excluded from analysis when the FD mean >0.5 mm. There were no significant differences in mean FD between PD patients (mean ± SD = 0.19 ± 0.076 mm) and HC (mean ± SD = 0.18 ± 0.079 mm) (*F* = 0.309; *p* = 0.579). Moreover, we included FD as a covariate in the neuroimaging analyses. In addition, we calculated the maximum displacement (maximum absolute value of displacement of each volume) in translation indexes x, y, or z was higher than 3.0 mm and in rotation, indexes was higher than 3.0°. One patient was excluded due to head motion. Therefore, the analyses were carried out with 99 PD patients and 62 HC. Moreover, during the fMRI preprocessing steps, the six motion parameters were regressed out with the anatomical CompCor approach to reduce the head motion and noise influence from the signal.

### Dynamic FC analysis

Time-varying FC analysis was examined with the dynamic functional network connectivity toolbox in GIFT. In order to analyze the temporal variations of dynamic FC, a sliding window approach was applied. A sliding time window of 22 TR with a size of 44 s was specified for each subject, with a Gaussian window alpha value of 3, and a step between windows of 1 TR. The window size was chosen following previous studies in PD for later results comparisons^16,19^ and following the previous recommendations^9^. Within each window, the time series data of the 53 ICs was used to obtain the 53 × 53 covariance matrices. Due to the short time segments that could have insufficient information, the regularized inverse covariance matrix was used^66^. Additionally, we specified the L1 regularization and repeated 100 times to promote sparsity^67^. Values in the FC matrices were converted into z-scores using Fisher’s z transformation to improve the normality of the distribution.

All the FC windows across all subjects were used to estimate the dynamic FC States (i.e., reoccurring FC patterns in several windows). We applied the k-means clustering method to cluster the FC windows. To estimate the similarities between each FC window and the cluster centroid (i.e., center), the L1 distance norm (Manhattan distance) was applied and was repeated 100 times to obtain the unbiased initial cluster. Windows consisting of local maxima in FC variance were used to reduce redundancy between windows as well as computational demand^9^. To estimate the optimal number of FC States, we performed clustering validity with gap and silhouette criteria. Both methods determined the number of clusters (i.e., States) (k = 2). After that, all windows across all subjects were classified and categorized in each dynamic FC State, following the similarities with each cluster centroid. The final cluster centroids were calculated with the median of all the regrouped FC windows in each State (Fig. 2). For each subject, a subject-specific centroid was calculated, with the median value of each FC matrix for that State. Figure 3 shows the mean matrix per each State for each PD and HC male and female groups. Supplementary Fig. 2 shows the group-specific mean per each State for PD subgroups and HC.

The following temporal features of the FC States were analyzed for each subject: (1) *Fractional time window* defined as the total time spent in one State (measured in percentage); (2) *Mean dwell time* defined as the number of consecutive windows in a specific State, before switching to another State (measured in windows); and (3) Number of transitions between States or *State transition* was calculated counting the total number of changes between States^9^.

### Hierarchical clustering analysis in PD

To answer the second objective and accurately classify the patients based on motor and non-motor symptoms, we used hierarchical agglomerative clustering analysis performed in Statistical Package for Social Science (SPSS) (IBM SPSS Statistics 22). The clustering analysis first starts with each subject separately, and at each step combines subjects by pairs based on similarities, until the last two clusters combined include all the participants. The clustering method used was Ward’s clustering linkage method with squared Euclidean distance. This method minimizes the sum of square errors from the cluster mean^68^.

The variables introduced for clustering analysis were the UPDRS III, Hoehn and Yahr stage, MoCA for cognitive impairment, and GDS-15 for depressive symptoms. All variables were introduced in *z*-scores. Different numbers of clusters were specified (2–4) to evaluate the more accurate distinction between patients. Among the solutions, 2 clusters only made a distinction between lower or higher motor symptoms but not a distinction in non-motor symptoms. The 4-cluster solution included one group too small for statistical analyses (*n* = 12). The three-cluster solution showed significant differences in motor and non-motor symptomatology. Therefore, the optimal number of clusters for our PD group was set to three because of the better distribution and clinical relevance (Fig. 3).

PCA was performed to validate the PD subgroup classification obtained from the clustering analysis. PCA transforms data into linear components, with the objective of reducing the number of variables in components that explain the maximum total variance^69^. The four variables were introduced in the analysis and two components were obtained: a motor component and a non-motor component. The combination of these two components could be interpreted as the best representation of the variability and distribution of the data (Supplementary Fig. 1).

### Statistical analysis

Statistical analyses were performed in the SPSS (IBM SPSS Statistics 22). Sociodemographic and clinical differences were calculated with Analysis of Variance (ANOVA) within each group (PD and HC). Cognitive and depression differences were calculated with two-way MANOVA for between-group comparisons, to test GroupxSex interaction.

The first objective of the study was to evaluate the influence of sex on dynamic FC characteristics both in HC and PD patients. To answer this first objective, we performed between-group comparisons with two-way MANCOVA to assess GroupxSex interaction with acquisition site, FD mean, age, years of education, MoCA score, and depression score as covariates. Then, within-group comparisons were analyzed with the same covariates.

To answer the second objective, we used hierarchical agglomerative clustering analysis in SPSS to classify the patients based on their symptomatology. Sociodemographic, clinical, and cognitive/behavioral differences between PD subgroups and HC were assessed with one-way ANOVA. Chi-squared analysis was used to determine significant sex differences in the frequency of the PD subgroups and HC. Dynamic FC differences between PD subgroups and HC were assessed with one-way MANCOVA with acquisition site, FD mean, and age as covariates in these secondary analyses. Additionally, because LEDD was found to influence brain FC in PD^70^, LEDD was introduced as a covariate for all rs-fMRI data. Results are shown with *p* < 0.05 (two-tailed). Moreover, FC differences within each State between groups were calculated (*p* < 0.05 FDR corrected). Finally, Pearson’s correlation analyses were carried out between temporal properties of connectivity and PD symptomatology. To do so, we created a PD symptomatology variable with a composite score of the UPDRS III, Hoehn & Yahr, MoCA, and depression scores. MoCA scores were recoded so that higher values represented greater cognitive impairment. RStudio v1.3.1093 was used for results visualization.

### Reporting Summary

Further information on research design is available in the Nature Research Reporting Summary linked to this article.

## Supplementary information

Supplementary Information

Reporting Summary

## Data Availability

The data that support the findings of this study are available from the corresponding author upon reasonable request and once the project is finalized.

## References

[1] Baggio H (2015). Cognitive impairment and resting-state network connectivity in Parkinson’s disease. Hum. Brain Mapp..

[2] Wolters AF (2018). Resting-state fMRI in Parkinson’s disease patients with cognitive impairment: a meta-analysis. Parkinsonism Relat. Disord..

[3] Strafella AP (2018). Imaging markers of progression in Parkinson’s disease. Mov. Disord. Clin. Pract..

[4] Sheng K (2014). Altered spontaneous brain activity in patients with Parkinson’s disease accompanied by depressive symptoms, as revealed by regional homogeneity and functional connectivity in the prefrontal-limbic system. PLoS ONE.

[5] Lou Y (2015). Altered brain network centrality in depressed Parkinson’s disease patients. Mov. Disord..

[6] Hu X (2015). Abnormal functional connectivity of the amygdala is associated with depression in Parkinson’s disease. Mov. Disord..

[7] Wei L (2017). Aberrant intra-and internetwork functional connectivity in depressed Parkinson’s disease. Sci. Rep..

[8] Hutchison RM (2013). Dynamic functional connectivity: promise, issues, and interpretations. NeuroImage.

[9] Allen EA (2014). Tracking whole-brain connectivity dynamics in the resting state. Cereb. cortex.

[10] Calhoun VD, Miller R, Pearlson G, Adalı T (2014). The chronnectome: time-varying connectivity networks as the next frontier in fMRI data discovery. Neuron.

[11] Kaiser RH (2016). Dynamic resting-state functional connectivity in major depression. Neuropsychopharmacology.

[12] Demirtaş M (2016). Dynamic functional connectivity reveals altered variability in functional connectivity among patients with major depressive disorder. Hum. Brain Mapp..

[13] Damaraju E (2014). Dynamic functional connectivity analysis reveals transient states of dysconnectivity in schizophrenia. NeuroImage Clin..

[14] Du Y (2016). Interaction among subsystems within default mode network diminished in schizophrenia patients: a dynamic connectivity approach. Schizophrenia Res..

[15] Jones DT (2012). Non-stationarity in the “resting brain’s” modular architecture. PLoS ONE.

[16] Kim J (2017). Abnormal intrinsic brain functional network dynamics in Parkinson’s disease. Brain.

[17] d’Ambrosio A (2019). Reduced dynamics of functional connectivity and cognitive impairment in multiple sclerosis. Mult. Scler. J..

[18] Fu Z (2019). Altered static and dynamic functional network connectivity in Alzheimer’s disease and subcortical ischemic vascular disease: shared and specific brain connectivity abnormalities. Hum. Brain Mapp..

[19] Fiorenzato E (2019). Dynamic functional connectivity changes associated with dementia in Parkinson’s disease. Brain.

[20] Díez-Cirarda M (2017). Dynamic functional connectivity in Parkinson’s disease patients with mild cognitive impairment and normal cognition. NeuroImage Clin..

[21] Schapira AHV, Chaudhuri KR, Jenner P (2017). Non-motor features of Parkinson disease. Nat. Rev. Neurosci..

[22] Georgiev D, Hamberg K, Hariz M, Forsgren L, Hariz G-M (2017). Gender differences in Parkinson’s disease: a clinical perspective. Acta Neurologica Scandinavica.

[23] Miller IN, Cronin‐Golomb A (2010). Gender differences in Parkinson’s disease: clinical characteristics and cognition. Mov. Disord..

[24] Reekes TH (2020). Sex specific cognitive differences in Parkinson disease. npj Parkinson’s Dis..

[25] Augustine EF (2015). Sex differences in clinical features of early, treated Parkinson’s disease. PLoS ONE.

[26] De Micco R (2019). Sex-related pattern of intrinsic brain connectivity in drug-naïve Parkinson’s disease patients. Mov. Disord..

[27] Biswal BB (2010). Toward discovery science of human brain function. Proc. Natl Acad. Sci. USA.

[28] Allen EA (2011). A baseline for the multivariate comparison of resting-state networks. Front. Syst. Neurosci..

[29] Ritchie SJ (2018). Sex differences in the adult human brain: evidence from 5216 UK Biobank participants. Cereb. Cortex.

[30] Zhang C, Dougherty CC, Baum SA, White T, Michael AM (2018). Functional connectivity predicts gender: evidence for gender differences in resting brain connectivity. Hum. Brain Mapp..

[31] de Lacy N, McCauley E, Kutz JN, Calhoun VD (2019). Sex-related differences in intrinsic brain dynamism and their neurocognitive correlates. NeuroImage.

[32] Gur RC, Gur RE (2017). Complementarity of sex differences in brain and behavior: from laterality to multimodal neuroimaging. J. Neurosci. Res..

[33] Scheinost D (2015). Sex differences in normal age trajectories of functional brain networks. Hum. Brain Mapp..

[34] Mars R (2012). On the relationship between the “default mode network” and the “social brain”. Front. Hum. Neurosci..

[35] Wirth M (2011). Semantic memory involvement in the default mode network: a functional neuroimaging study using independent component analysis. NeuroImage.

[36] Cassady K (2019). Sensorimotor network segregation declines with age and is linked to GABA and to sensorimotor performance. NeuroImage.

[37] Albert NB, Robertson EM, Miall RC (2009). The resting human brain and motor learning. Curr. Biol..

[38] Haaxma CA (2007). Gender differences in Parkinson’s disease. J. Neurol. Neurosurg. Psychiatry.

[39] Sauerbier A, Jenner P, Todorova A, Chaudhuri KR (2016). Non motor subtypes and Parkinson’s disease. Parkinsonism Relat. Disord..

[40] Mallol R (2007). Compensatory cortical mechanisms in Parkinson’s disease evidenced with fMRI during the performance of pre-learned sequential movements. Brain Res..

[41] Sabatini U (2000). Cortical motor reorganization in akinetic patients with Parkinson’s disease: a functional MRI study. Brain.

[42] Wu T (2009). Regional homogeneity changes in patients with Parkinson’s disease. Hum. Brain Mapp..

[43] Göttlich M (2013). Altered resting state brain networks in Parkinson’s disease. PLoS ONE.

[44] Laufs H (2003). Electroencephalographic signatures of attentional and cognitive default modes in spontaneous brain activity fluctuations at rest. Proc. Natl Acad. Sci. USA.

[45] Silberstein P (2005). Cortico-cortical coupling in Parkinson’s disease and its modulation by therapy. Brain.

[46] Stoffers D (2008). Increased cortico-cortical functional connectivity in early-stage Parkinson’s disease: an MEG study. NeuroImage.

[47] Kovács M (2016). Impact of sex on the nonmotor symptoms and the health-related quality of life in Parkinson’s disease. Parkinson’s Dis..

[48] Litvan I (2011). MDS task force on mild cognitive impairment in Parkinson’s disease: critical review of PD-MCI. Mov. Disord..

[49] Pagonabarraga J, Kulisevsky J, Strafella AP, Krack P (2015). Apathy in Parkinson’s disease: clinical features, neural substrates, diagnosis, and treatment. Lancet Neurol..

[50] Reuter M (2015). Head motion during MRI acquisition reduces gray matter volume and thickness estimates. NeuroImage.

[51] Backhausen LL (2016). Quality control of structural MRI images applied using FreeSurfer—A hands-on workflow to rate motion artifacts. Front. Neurosci..

[52] Hoehn MM, Yahr MD (1967). Parkinsonism: onset, progression, and mortality. 1967. Neurology.

[53] Tomlinson CL (2010). Systematic review of levodopa dose equivalency reporting in Parkinson’s disease. Mov. Disord..

[54] Nasreddine ZS (2005). The Montreal cognitive assessment, MoCA: a brief screening tool for mild cognitive impairment. J. Am. Geriatrics Soc..

[55] Folstein MF, Robins LN, Helzer JE (1983). The mini-mental state examination. Arch. Gen. Psychiatry.

[56] Yesavage JA, Sheikh JI (1986). 9/Geriatric depression scale (GDS) recent evidence and development of a shorter violence. Clin. Gerontologist.

[57] Beck AT, Steer RA, Brown GK (1996). Beck depression inventory-II. San. Antonio.

[58] Bergeron D (2017). Multicenter validation of an MMSE‐Mo CA conversion table. J. Am. Geriatrics Soc..

[59] Ferreira D (2017). The interactive effect of demographic and clinical factors on hippocampal volume: a multicohort study on 1958 cognitively normal individuals. Hippocampus.

[60] Whitfield-Gabrieli S, Nieto-Castanon A (2012). Conn: a functional connectivity toolbox for correlated and anticorrelated brain networks. Brain Connectivity.

[61] Weissenbacher A (2009). Correlations and anticorrelations in resting-state functional connectivity MRI: a quantitative comparison of preprocessing strategies. NeuroImage.

[62] Calhoun VD, Adali T, Pearlson GD, Pekar JJ (2001). A method for making group inferences from functional MRI data using independent component analysis. Hum. Brain Mapp..

[63] Himberg J, Hyvärinen A, Esposito F (2004). Validating the independent components of neuroimaging time series via clustering and visualization. NeuroImage.

[64] Shirer WR, Ryali S, Rykhlevskaia E, Menon V, Greicius MD (2012). Decoding subject-driven cognitive states with whole-brain connectivity patterns. Cereb. cortex.

[65] Power JD, Barnes KA, Snyder AZ, Schlaggar BL, Petersen SE (2012). Spurious but systematic correlations in functional connectivity MRI networks arise from subject motion. NeuroImage.

[66] Varoquaux, G., Gramfort, A., Poline, J.-B. & Thirion, B. Brain covariance selection: better individual functional connectivity models using population prior. *Adv. Neural Inf. Process. Syst.* 2334–2342 (2010).

[67] Friedman J, Hastie T, Tibshirani R (2008). Sparse inverse covariance estimation with the graphical lasso. Biostatistics.

[68] Ward JH (1963). Hierarchical grouping to optimize an objective function. J. Am. Stat. Assoc..

[69] Field, A. *Discovering Statistics Using IBM SPSS Statistics* (Sage, 2013).

[70] Mattay VS (2002). Dopaminergic modulation of cortical function in patients with Parkinson’s disease. Ann. Neurol..

