# Supplementary material for: Contributions of sex, depression, and cognition on brain connectivity dynamics in Parkinson’s disease
Source: NPJ Parkinsons Dis. 2021 Dec 16;7:117. doi: 10.1038/s41531-021-00257-9 (PMC8677758; doi:10.1038/s41531-021-00257-9)
Supplement: Supplementary file 1 — Supplementary Information [file 41531_2021_257_MOESM1_ESM.pdf]

Supplementary Materials

Contributions of sex, depression, and cognition on brain connectivity dynamics in Parkinson's disease

Maria Diez-Cirarda[1,2,3*], Iñigo Gabilondo[3,4], Naroa Ibarretxe-Bilbao[5], Juan Carlos Gómez-Esteban[3,6], Jinhee Kim[1,2], Olaia Lucas-Jiménez[5], Rocio del Pino[3], Javier Peña[5], Natalia Ojeda[5], Alexander Mihaescu[1,2], Mikaeel Valli[1,2], Maria Angeles Acera[3], Alberto Cabrera-Zubizarreta[7], Maria Angeles Gómez-Beldarrain[8], and Antonio P. Strafella[1,2**]

1- Research Imaging Centre, Campbell Family Mental Health Research Institute, Centre for Addiction and Mental Health, University of Toronto, Toronto (Canada)
2- E.J. Safra Parkinson Disease Program & Movement Disorder Unit, Neurology Division; Krembil Brain Institute, University Health Network, University of Toronto, Toronto (Canada)
3- Neurodegenerative Diseases Group, Biocruces Bizkaia Health Research Institute, Barakaldo (Spain)
4- IKERBASQUE, The Basque Foundation for Science, Bilbao (Spain)
5- Department of Methods and Experimental Psychology, University of Deusto, Bilbao (Spain)
6- Neurology Department, Cruces University Hospital, Barakaldo, (Spain)
7- OSATEK, MR Unit, Hospital of Galdakao, Galdakao, Bilbao, (Spain)
8- Neurology Service, Hospital of Galdakao, Galdakao, Bilbao (Spain)

*Corresponding author: email: maria.diecirarda@gmail.com (Dr. Maria Díez-Cirarda)
**Corresponding author: mail: Antonio.Strafella@camh.ca (Dr. AP Strafella)

1. Supplementary Tables

1.1 Supplementary Table 1: MRI acquisition parameters for each acquisition site

1.2 Supplementary Table 2: Peak coordinates of the selected Independent Components (ICs=53)

2. Supplementary Figures

2.1. Supplementary Figure 1: Principal Component Analysis

2.2. Supplementary Figure 2: Dynamic FC States in PD subgroups and HC

2.3. Supplementary Figure 3: Schematic representation of neuroimaging preprocessing and analyses

2.4. Supplementary Figure 4: Principal Component Analysis with PD males and females distribution.

3. Supplementary Results:

3.1. Sex differences within each PD subgroup: Motor and non-motor symptoms

3.2. Sex differences within each PD subgroup: Dynamic FC indexes

Supplementary Tables

Supplementary Table 1: MRI acquisition parameters for each acquisition site

	**Research Imaging Centre (CAMH) Toronto (Canada)**	**BioCruces Research Institute & Cruces Hospital Bizkaia (Spain)**	**University of Deusto Bizkaia (Spain)**
**Sample**	PD = 37; HC = 16	PD = 40; HC = 21	PD = 22; HC = 25
**Scanner type**	3T MRI scanner General Electric Discovery MR750	3T MRI scanner Philips Achieva TX	3T MRI scanner Philips Achieva TX
**MRI acquisition parameters**			
T1			
	Fast Spoiled Gradient Echo pulse sequence	Multi-Slice Gradient echo EPI sequence	Multi-Slice Gradient echo EPI sequence
**Repetition time (TR)**	6.7 ms	7.4 ms	7.4 ms
**echo time (TE)**	3.0 ms	3.4 ms	3.4 ms
**Flip angle**	8°	9°	9°
**Field of view (FoV)**	230 × 230mm^2	250 × 250 mm^2	250 × 250mm^2
**Matrix size**	256 × 256	228 × 218	228 × 218
**Slices**	sagittal	sagittal	sagittal
**Slice thickness**	0.9 mm	1.1 mm	1.1 mm
**Num. slices**	200 slices	300 slices	300 slices
**Voxel size**	0.89 × 0.89 × 0.9mm^3	0.98 × 0.98 × 0.60 mm^3	0.98 × 0.98 × 0.60 mm^3
Rs-fMRI			
**Repetition time (TR)**	2000 ms	2100 ms	2100 ms
**Echo time (TE)**	30 ms	27 ms	16 ms
**Flip angle**	60°	80°	80°
**Field of view (FoV)**	220 × 220 mm^2	240 × 240 mm^2	240 × 240mm^2
**Matrix size**	64 × 64	80 × 79	80 ×78
**Num. slices**	31	45	40
**Slice thickness**	5 mm	3 mm	3 mm
**Slice order**	Interleave botton-up	Interleave botton-up	Interleave botton-up
**Gap**	no gap	0.25 mm	0.25 mm
**Num. Volumes**	242 (240 vol analyzed)	217 (214 vol analyzed)	217 (214 vol analyzed)
**Voxel size**	3.43 × 3.43 × 5.0 mm^3	3.00 × 3.00 × 3.00 mm^3	3.00 × 3.00 × 3.00 mm^3
**Acquisition time**	8’4’’	7’40’’	7’40’’

Supplementary Table 2: Peak coordinates of the selected Independent Components (ICs=53)

Intrinsic Connectivity Networks		BA	t_{max}	Peak coordinate (mm)		
				x	y	z
Subcortical Network (SC, 7)						
IC9	Caudate		38.57	-11	-3	13
IC13	Putamen		28.44	18	12	-8
IC26	Caudate		40.85	9	17	2
IC33	Thalamus		31.82	14	-21	7
IC34	Putamen		35.77	25	4	1
IC44	Red Nucleus		34.05	7	-16	-9
IC87	Putamen		24.90	-27	-6	-6
Auditory Network (AUD, 4)						
IC30	Superior Temporal Gyrus	41	34.53	51	-18	2
IC48	Supramarginal Gyrus, Parietal Lobe	40	27.71	-47	-22	18
IC59	Middle Temporal Gyrus	21	27.60	66	-22	-5
IC61	Supramarginal Gyrus, Parietal Lobe	40	29.54	54	-30	21
Somatomotor Network (SM, 7)						
IC2	Primary Motor Cortex	4	38.64	4	-25	65
IC3	Primary Motor Cortex	4	54.92	56	-6	29
IC6	Precentral Gyrus	4	38.53	-36	-25	53
IC8	Precentral Gyrus	4	37.41	39	-21	56
IC14	Supplementary Motor Cortex	6	28.31	16	-10	72
IC28	Superior Parietal Lobe	7	31.59	17	-53	67
IC45	Premotor Cortex	6	33.22	-7	0	47
Visual Network (VIS, 9)						
IC5	Lingual Gyrus	18	36.58	27	-97	-4
IC15	Cuneus	18	36.02	3	-81	26
IC32	Lingual Gyrus	18	33.39	18	-75	-5
IC37	Cuneus	17	36.75	6	-90	8
IC40	Visual Association Cortex	18	29.41	-30	-81	6
IC73	Primary Visual Cortex	17	26.02	16	-65	5
IC85	Superior Occipital Gyrus	19	33.56	39	-81	31
IC88	Visual Association Cortex	18	27.82	33	-80	6
IC93	Culmen	37	31.02	34	-48	-18
Cognitive Executive Network (CEN, 13)						
IC12	Superior Frontal Gyrus	6	35.18	-9	12	65
IC35	Inferior Frontal Gyrus	45	28.14	-55	30	4
IC41	Insula	13	33.21	44	1	-4
IC43	Anterior Prefrontal Cortex	10	30.00	-28	48	15
IC49	Middle Temporal Gyrus	38	23.55	-34	8	-38
IC51	Medial Prefrontal Cortex	9	29.82	43	19	27
IC58	Insula	13	31.18	-32	21	1

(continued on next page)

Intrinsic Connectivity Networks		BA	t_{max}	Peak coordinate (mm)		
				x	y	z
IC75	Precentral Gyrus	6	23.10	-44	-6	48
IC77	Insula	13	31.35	42	0	11
IC83	Inferior Parietal Lobe	40	33.43	43	-36	42
IC86	Inferior Parietal Lobe	40	32.58	-60	-28	30
IC89	Superior Parietal Lobe	7	31.86	-27	-67	45
IC98	Fusiform Gyrus	37	25.30	-51	-49	-10
Default-Mode Network (DMN, 10)						
IC21	Anterior Cingulate Cortex	24	34.82	-1	31	18
IC23	Dorsal Anterior Cingulate Area	32	29.89	-9	39	7
IC38	Orbitofrontal Cortex	11	35.56	9	32	-10
IC50	Superior Temporal Gyrus	22	34.06	47	-43	10
IC60	Superior Frontal Gyrus	8	27.46	8	41	43
IC70	Angular gyrus, Parietal Lobe	39	36.22	50	-61	35
IC72	Inferior Frontal Gyrus	45	28.11	52	30	1
IC91	Angular gyrus, Parietal Lobe	39	34.27	-54	-56	33
IC97	Precuneus	7	42.61	-6	-70	35
IC100	Angular gyrus, Parietal Lobe	39	25.21	-44	-74	34
Cerebellar Network (CB, 3)						
IC31	Cerebellar Tonsil		22.21	29	-55	-35
IC76	Pyramis		28.68	33	-78	-34
IC78	Cerebellar Tonsil		29.10	-33	-60	-35

Coordinates represent the peak voxel of each IC (based on one sample t-test for each component.
BA= Brodmann Area; IC= Independent Component.

Supplementary Figures

Supplementary Figure 1: Principal Component Analysis

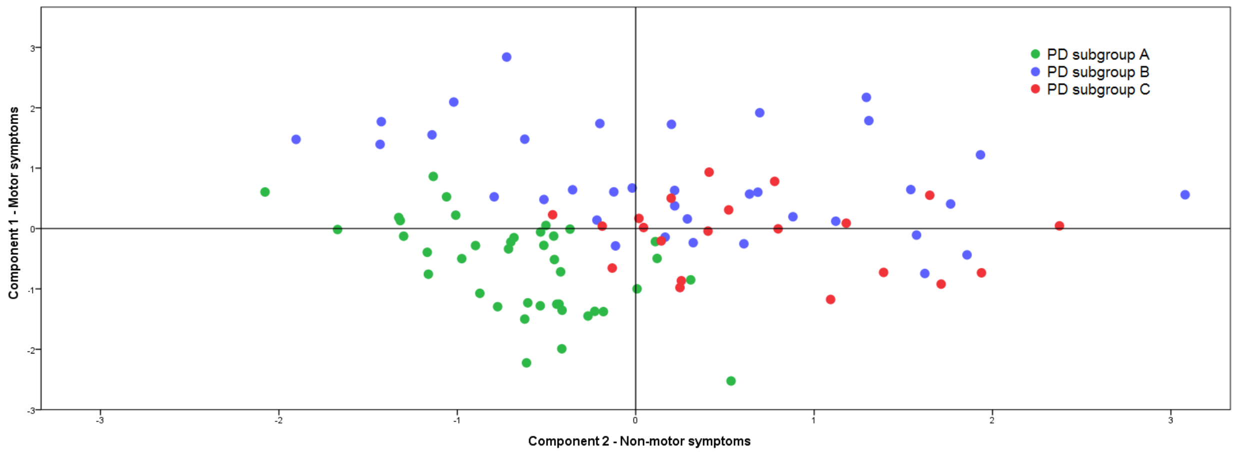

Supplementary Figure 1 Legend: *X*-axis represents non-motor symptomatology. Values represent a combination of presence or absence of cognitive or depressive symptoms. *Y*-axis represents motor symptomatology. Values > 0 represent high motor symptoms, and Values < 0 represent low motor symptoms. The first component represents motor symptoms (i.e. UPDRS III and Hoehn & Yahr), that explains the 43.27% of the variance, and the second component represents non-motor symptoms (i.e. MoCA scores and Depression scores) and explains the 24.87% of the variance.

Supplementary Figure 2: Dynamic FC States in PD subgroups and HC

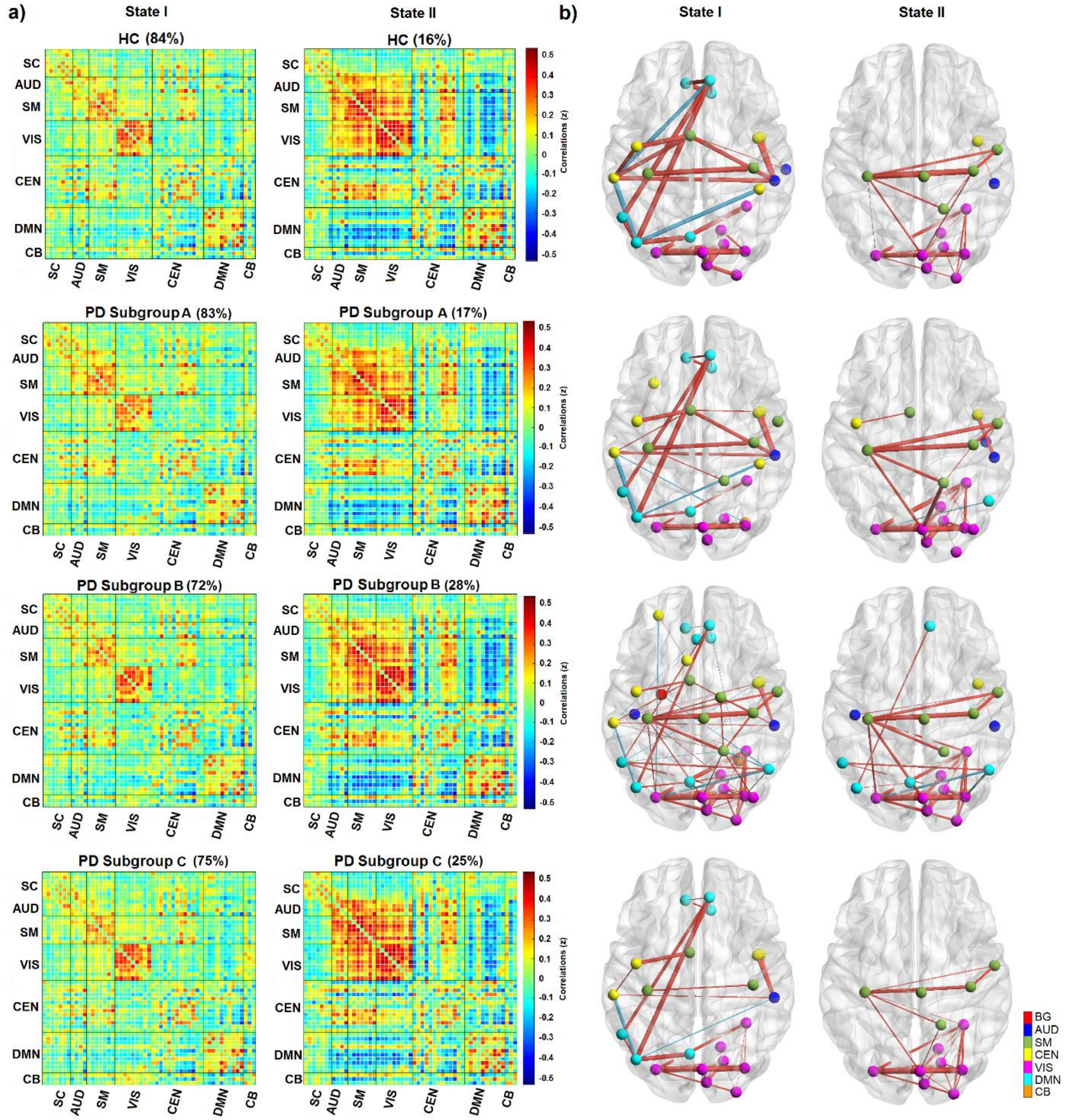

Supplementary Figure 2 Legend: a) Mean FC State per group. Percentage indicates occurrence rate; b) 2% of the strongest connections of each group averaged FC matrix. Red connections indicate positive correlation; Blue connections indicate negative correlation.

Supplementary Figure 3: Schematic representation of neuroimaging preprocessing and analyses

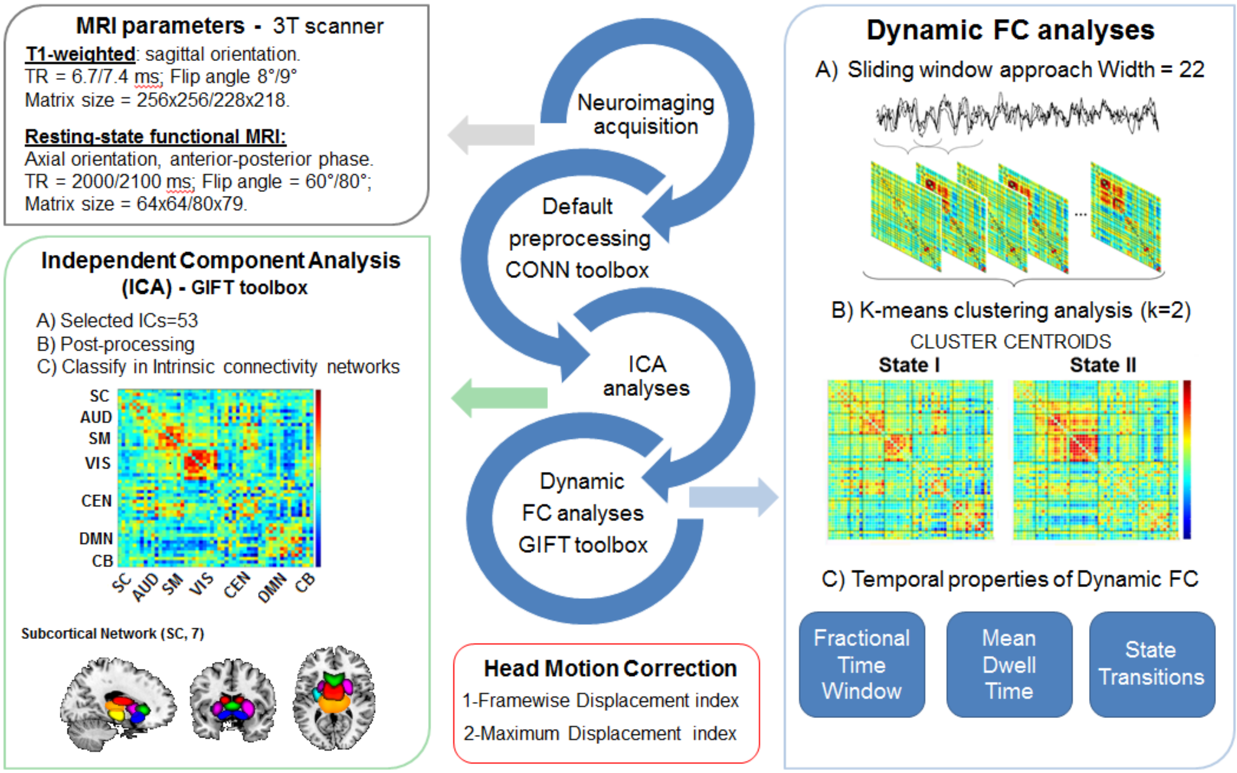

Supplementary Figure 4: Principal Component Analysis with PD males and females distribution.

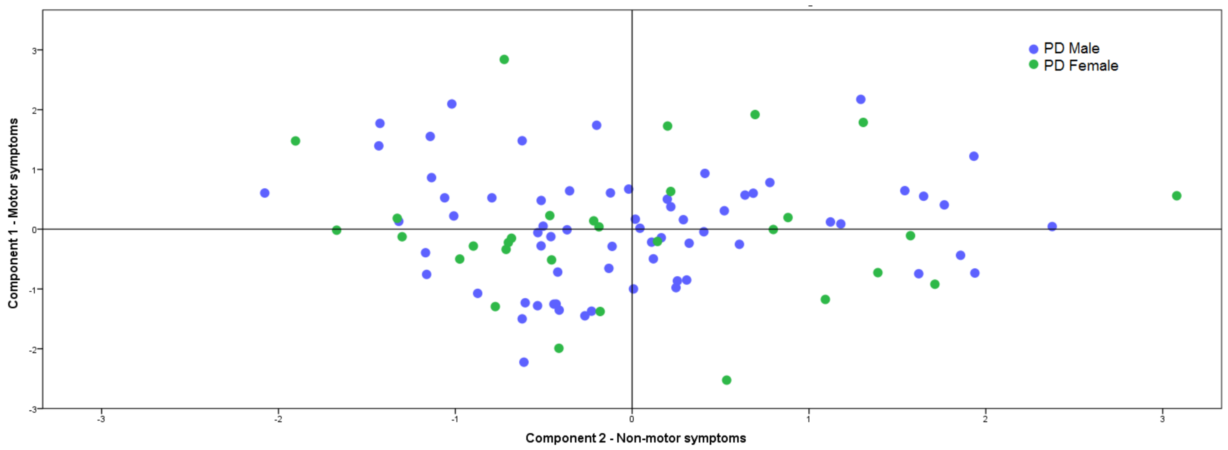

Supplementary Figure 4 Legend: *X*-axis represents non-motor symptomatology. Values > 0 represent high motor symptoms, and Values < 0 represent low motor symptoms; *Y*-axis represents motor symptomatology. Values represent a combination of presence or absence of cognitive or depressive symptoms.

Supplementary Results

3. Sex differences within each PD subgroup

3.1. Sex differences within each PD subgroup: Motor and non-motor symptoms

As additional analyses, we evaluated motor and non-motor differences between males and females in each PD subgroup and found no significant differences. Males and females in PD subgroup A showed no significant differences in Hoehn & Yahr (p = .789), disease duration (p = .831), UPDRS III (p = .148), MoCA (p = .593), depression score (p = .880) or LEDD (p = .867). Additionally, males and females in PD subgroup B showed no significant differences: Hoehn & Yahr (p = .084), disease duration (p = .158), UPDRS III (p = .243), MoCA (p = .257), depression score (p = .636) or LEDD (p = .403). Moreover, males and females in PD subgroup C showed no significant differences in Hoehn & Yahr (p = .715), disease duration (p = .848), UPDRS III (p = .221), MoCA (p = .075), depression score (p = .829) or LEDD (p = .195). Supplementary Figure 4 shows males and females equally distributed across the PCA analysis with the two components (motor and non-motor symptoms).

3.2. Sex differences within each PD subgroup: Dynamic FC indexes

In addition, we analyzed the sex differences in dynamic indexes within each PD subgroup, and we found no significant differences in any of the PD subgroups A, B or C. PD subgroup A showed no significant differences between males and females in *fractional time window* (p = .201), *mean dwell time* State I (p = .434) or *state transitions* (p = .362). PD subgroup B revealed no significant differences between males and females in *fractional time window* (p = .862), *mean dwell time* State I (p = .526) or *state transitions* (p = .323). PD subgroup C revealed no significant differences between males and females in *fractional time window* (p = .521), *mean dwell time* State

I (p = .339) or *state transitions* (p = .167).
