# Supplementary material for: Contributions of sex, depression, and cognition on brain connectivity dynamics in Parkinson’s disease
Source: NPJ Parkinsons Dis. 2021 Dec 16;7:117. doi: 10.1038/s41531-021-00257-9 (PMC8677758; doi:10.1038/s41531-021-00257-9)
Supplement: Supplementary file 2 — Reporting Summary [file 41531_2021_257_MOESM2_ESM.pdf]

nature research

Corresponding author(s): Dr. Diez-Cirarda and Dr. Strafella

Last updated by author(s): Jan 25, 2021

Reporting Summary

Nature Research wishes to improve the reproducibility of the work that we publish. This form provides structure for consistency and transparency in reporting. For further information on Nature Research policies, see our Editorial Policies and the Editorial Policy Checklist.

Statistics

For all statistical analyses, confirm that the following items are present in the figure legend, table legend, main text, or Methods section.

n/a	Confirmed	
☐	☒	The exact sample size (n) for each experimental group/condition, given as a discrete number and unit of measurement
☐	☒	A statement on whether measurements were taken from distinct samples or whether the same sample was measured repeatedly
☐	☒	The statistical test(s) used AND whether they are one- or two-sided<br>*Only common tests should be described solely by name; describe more complex techniques in the Methods section.*
☐	☒	A description of all covariates tested
☐	☒	A description of any assumptions or corrections, such as tests of normality and adjustment for multiple comparisons
☐	☒	A full description of the statistical parameters including central tendency (e.g. means) or other basic estimates (e.g. regression coefficient) AND variation (e.g. standard deviation) or associated estimates of uncertainty (e.g. confidence intervals)
☐	☒	For null hypothesis testing, the test statistic (e.g. F, t, r) with confidence intervals, effect sizes, degrees of freedom and P value noted<br>*Give P values as exact values whenever suitable.*
☒	☐	For Bayesian analysis, information on the choice of priors and Markov chain Monte Carlo settings
☐	☒	For hierarchical and complex designs, identification of the appropriate level for tests and full reporting of outcomes
☒	☐	Estimates of effect sizes (e.g. Cohen's d, Pearson's r), indicating how they were calculated

Our web collection on statistics for biologists contains articles on many of the points above.

Software and code

Policy information about availability of computer code

Data collection	no software was used.
Data analysis	CONN toolbox 18.a, Group ICA of fMRI Toolbox (GIFT) v4.0b, Statistical Package for the Social Sciences (SPSS) v22.0.

For manuscripts utilizing custom algorithms or software that are central to the research but not yet described in published literature, software must be made available to editors and reviewers. We strongly encourage code deposition in a community repository (e.g. GitHub). See the Nature Research guidelines for submitting code & software for further information.

Data

Policy information about availability of data

All manuscripts must include a data availability statement. This statement should provide the following information, where applicable:

- Accession codes, unique identifiers, or web links for publicly available datasets
- A list of figures that have associated raw data
- A description of any restrictions on data availability

The data that support the findings of this study are available from the corresponding author upon reasonable request and once the project is finalized.

Field-specific reporting

Please select the one below that is the best fit for your research. If you are not sure, read the appropriate sections before making your selection.

☐ Life sciences ☒ Behavioural & social sciences ☐ Ecological, evolutionary & environmental sciences

For a reference copy of the document with all sections, see nature.com/documents/nr-reporting-summary-flat.pdf

Behavioural & social sciences study design

All studies must disclose on these points even when the disclosure is negative.

Study description	cross-sectional study with quantitative data
Research sample	The sample was composed by Parkinson´s disease participants and healthy controls. This study included a retrospective pooled database from three research centers: the Biocruces Bizkaia Health Research Institute (Barakaldo, Spain), the University of Deusto (Bilbao, Spain) and the Centre for Addiction and Mental Health (Toronto, Canada). Healthy controls were matched by age, sex and education with Parkinson´s disease participants.
Sampling strategy	Sample was pooled from the three collaborative research centers.
Data collection	Data were collected in paper and pencil for sociodemographics, neuropsychological and clinical assessments and then introduced in SPSS for analysis. fMRI was also aquired.
Timing	This is a collaborative study from three research centers: Centre for Addiction and Mental Health (start: 2013- end: 2015) ; BioCruces health Research Institute (Start: 2015-end:2017); University of Deusto (Start: 2012-end:2016)
Data exclusions	After reviewing MRI images for quality control, twenty-four participants were excluded. In addition, two HC were excluded due to enlarged ventricles. Nine participants were excluded due to participation in the two Spanish research centers, which are in the same province. Database included in this study was 100 PD patients and 62 HC. After motion correction, final sample size was 99 PD patients and 62 HC.
Non-participation	no participants dropped out.
Randomization	Participants were not allocated into experimental groups.

Reporting for specific materials, systems and methods

We require information from authors about some types of materials, experimental systems and methods used in many studies. Here, indicate whether each material, system or method listed is relevant to your study. If you are not sure if a list item applies to your research, read the appropriate section before selecting a response.

Materials & experimental systems

n/a	Involved in the study	
☒	☐	Antibodies
☒	☐	Eukaryotic cell lines
☒	☐	Palaeontology and archaeology
☒	☐	Animals and other organisms
☐	☒	Human research participants
☒	☐	Clinical data
☒	☐	Dual use research of concern

Methods

n/a	Involved in the study	
☒	☐	ChIP-seq
☒	☐	Flow cytometry
☐	☒	MRI-based neuroimaging

Human research participants

Policy information about studies involving human research participants

Population characteristics	see above
Recruitment	Participants were recruited from the Department of Neurology at hospitals from each research center, and from the Parkinson´s disease Biscay Association (ASPARBI) after providing informed consent.
Ethics oversight	Centre for Addiction and Mental Health

Note that full information on the approval of the study protocol must also be provided in the manuscript.

Magnetic resonance imaging

Experimental design

Design type: resting-state fMRI

Design specifications: resting-state fMRI was aquired in one single session, and duration was: 8´4´´ - 7´40´´

Behavioral performance measures: no tasks.

Acquisition

Imaging type(s): functional and structural

Field strength: 3T

Sequence & imaging parameters: Two research centers used Multi-Slice Gradient echo EPI sequence and the other center used Fast Spoiled Gradient Echo pulse sequence. T1-weighted: Field of view: 230x230mm2-250x250mm2. Matrix size: 256x256-228x218; Slice thickness: 0.9mm- 1.1mm; orientation: sagittal; TE: 3.0ms - 3.4ms. TR: 6.7 ms - 7.4ms. Flip angle: 8º - 9º.
Rs-fMRI: Field of view: 220x220mm2-240x240mm2. matrix size:64x64-80x79-80x78. slice thickness: 5mm-3mm. orientation: interleave botton-up. TE:30ms-27ms-16ms. TR:2000ms-2100ms.flip angle: 60º-80º.

Area of acquisition: whole brain

Diffusion MRI: ☐ Used ☒ Not used

Preprocessing

Preprocessing software: CONN toolbox 18.a. All preprocessing steps were conducted using the default preprocessing pipeline, including realignment to the first volume, slice-timing correction (interleaved botton-up), co-registration to structural data, spatial normalization into the standard MNI space (Montreal Neurological Institute) and finally a smoothing of Gaussian kernel of 6 mm FWMH was applied. Moreover, noise was reduced via the anatomical CompCor approach, which extracts principal components from white matter and cerebrospinal fluid time series.

Normalization: see above

Normalization template: see above

Noise and artifact removal: To minimize the impact of head movement in the dynamic connectivity results, strict criteria were applied. Framewise displacement (FD) index was calculated following the published formula (Power et al., 2012). Subjects were excluded from analysis when the FD mean > 0.5 mm. Moreover, we included FD mean as covariate in neuroimaging analyses. In addition, we calculated the maximum displacement (maximum absolute value of displacement of each volume) in translation indexes x, y, or z was higher than 3.0 mm and in rotation indexes was higher than 3.0º. One patient was excluded due to head motion. Therefore, the analyses were carried out with 99 PD patients and 62 HC. Moreover, during the fMRI preprocessing steps, the six motion parameters were regressed out with the anatomical CompCor approach, to reduce the head motion and noise influence from signal.

Volume censoring: no volumes were censored.

Statistical modeling & inference

Model type and settings: Preprocessed images were introduced in Group ICA of fMRI Toolbox (GIFT) to decompose the data into intrinsic FC networks. 53 independent components were selected and classified in 7 networks. Then, time-varying FC analysis was examined with the dynamic functional network connectivity toolbox in GIFT. In order to analyze the temporal variations of dynamic FC, a sliding window approach was applied. After, we applied k-means clustering method to cluster the FC windows. FC in each dynamic State was calculated in GIFT toolbox and differences between groups were calculated with two-sample t tests. The temporal properties of dynamic connectivity were extracted and introduced in SPSS for analysis.
To answer this first objective we performed between-groups comparison with two-way MANCOVA to assess GroupxSex interaction with acquisition site, FD mean, age, years of education, MoCA score, and depression score as covariates. Then, within-groups comparisons were analyzed with same covariates to test sex effect. Dependent variables were dynamic FC indexes: functional time window, mean dwell time and state transtitions. To answer the second objective we used hierarchical agglomerative clustering analysis in SPSS to classify the patients based on their symptomatology and one-way MANCOVA was used with acquisition site, FD mean, and age as covariates to test differences between groups in dynamic indexes.

Effect(s) tested: see above

Specify type of analysis: ☒ Whole brain ☐ ROI-based ☐ Both

Statistic type for inference (See Eklund et al. 2016): see above

Correction | FDR correction can be selected in GIFT toolbox

Models & analysis

n/a	Involved in the study
☐	☒ Functional and/or effective connectivity
☒	☐ Graph analysis
☒	☐ Multivariate modeling or predictive analysis

Functional and/or effective connectivity | dynamic FC indexes: functional time window, mean dwell time and state transtitions.
